# Supplementary material for: The prognostic and clinicopathological significance of desmoglein 2 in human cancers: a systematic review and meta-analysis
Source: PeerJ. 2022 Mar 22;10:e13141. doi: 10.7717/peerj.13141 (PMC8957267; doi:10.7717/peerj.13141)
Supplement: Supplemental Information 1 — When omitting the included studies one by one, the result of meta-analysis did not dramatically change, showing that our result was stable and robust. [file peerj-10-13141-s001.docx]

Supplementary file 1. The table of sensitivity analysis. When omitting the included studies one by one, the result of meta-analysis did not dramatically change, showing that our result was stable and robust.

| Study omitted | HR | 95%CI | P value | I^2^ (%) |
| --- | --- | --- | --- | --- |
| Cai 2017 | 1.00 | 0.67-1.51 | 0.99 | 89 |
| Jin 2020 | 0.93 | 0.68-1.28 | 0.67 | 87 |
| Sun 2020 | 0.96 | 0.68-1.35 | 0.82 | 89 |
| Fang 2014 | 1.08 | 0.78-1.49 | 0.66 | 89 |
| Han 2018 | 0.96 | 0.69-1.33 | 0.79 | 89 |
| Ormanns 2015 | 1.04 | 0.73-1.47 | 0.84 | 90 |
| Xu 2020 | 1.15 | 0.85-1.55 | 0.37 | 86 |
| Yashiro 2006 | 1.12 | 0.92-1.54 | 0.47 | 88 |
| Chen 2018 | 1.06 | 0.73-1.53 | 0.77 | 88 |
| Qin 2020 | 0.96 | 0.70-1.31 | 0.78 | 89 |
