# Supplementary material for: The prognostic and clinicopathological significance of desmoglein 2 in human cancers: a systematic review and meta-analysis
Source: PeerJ. 2022 Mar 22;10:e13141. doi: 10.7717/peerj.13141 (PMC8957267; doi:10.7717/peerj.13141)

Supplementary file 2. The results of data mining in TCGA database also indicated that high DSG2 expression was associated poor prognosis in NSCLC

Data mining in TCGA

Raw counts of DSG2 expression data and corresponding clinical information from NSCLC were obtained from The Cancer Genome Atlas (TCGA) dataset (https://portal.gdc.cancer.gov/) in May 2021, in which the method of acquisition and application complied with the guidelines and policies. The survival analysis with log-rank test was also used to compare the survival difference between above two groups or more groups. For Kaplan–Meier (KM) curves, p-values and hazard ratio (HR) with 95% confidence interval (CI) were generated by log-rank tests and univariate COX proportional hazards regression. All analytical methods above and R packages were performed using R software version v4.0.3. Join.Cox package was used for data processing and analysis. PowerPoint and Excel are also used as tools in the processing of image and table data.

The principle of KM method is to first calculate the probability of patients who have lived through a certain period of time to live through the next period (survival probability), and then multiply the survival probability one by one, that is, the survival rate of the corresponding period of time. Kaplan-Meier survival analysis of the prognostic analysis of DSG2 in NSCLC in the TCGA was shown below. The X axis of the survival curve is the year, and the Y axis represents the survival probability. In order to reflect the effect of DSG2 expression on the survival of the whole population of patients with non-small cell lung cancer, it is not classified according to the stage of cancer, treatment, age, sex, race, smoking history and other clinical characteristics, but grouped according to the average expression of DSG2, and comprehensively analyzed the prognosis of lung squamous cell carcinoma (LUSC) and lung adenocarcinoma (LUAD). According to the median of gene expression, samples were divided into two groups: high expression group and low expression group. By the time of data downloaded, the expression of DSG2 had no significant effect on the 5-year survival rate of patients with non-small cell lung cancer, but the survival rate of 10 years or more in the low expression group was higher than that in the high expression group. Among the 395 patients who died, the average survival time was 2.52 years in the low expression group and 2.40 years in the high expression group. Among the 604 patients who survived, the average survival time was 2.57 years in the low expression group and 2.47 years in the high expression group. COX regression model is a semi-parametric regression model which takes survival outcome and survival time as dependent variables to analyze the influence of many factors on survival time at the same time. Hazard Ratio (HR) is the ratio of two hazard rates. In medical and public studies, hazard ratios are often used to express the hazard differences between the experimental group and the control group. HR>1 indicated that high DSG2 expression was a risk factor . P<0.05 was considered as statistically significant.


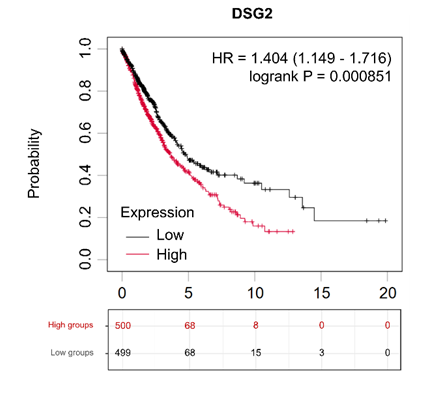

Supplement: Supplemental Information 2 [file peerj-10-13141-s002.docx]
