# Supplementary material for: The prognostic and clinicopathological significance of desmoglein 2 in human cancers: a systematic review and meta-analysis
Source: PeerJ. 2022 Mar 22;10:e13141. doi: 10.7717/peerj.13141 (PMC8957267; doi:10.7717/peerj.13141)
Supplement: Supplemental Information 4 [file peerj-10-13141-s004.docx]

1. The rationale for conducting the systematic review / meta-analysis

Answer:

It has been reported that aberrant expression of DSG2 was associated with tumorigenesis and cancer progression in a variety of human cancers. However, reports regarding the association between DSG2 expression and cancer prognosis were controversial. Until now, the relationship between DSG2 expression and prognosis in cancer patients has been inconclusive and not well reviewed. Therefore, this study aimed to explore the prognostic value of DSG2 in patients with cancer.

1. The contribution that it makes to knowledge in light of previously published related reports.

Answer:

A number of published clinical studies have researched the association between DSG2 expression and prognosis in different type of cancers, for example, (Cai et al. 2017; Jin et al. 2020; Sun et al. 2020) in respiratory system cancer, (Barber et al. 2014; Fang et al. 2014; Ormanns et al. 2015; Xu et al. 2020; Yashiro et al. 2006) in digestive system cancer and (Chen et al. 2018; Qin et al. 2020) in reproductive system cancer. However, no systematic review or meta-analysis has been published in his field.

References:

Barber AG, Castillo-Martin M, Bonal DM, Rybicki BA, Christiano AM, and Cordon-Cardo C. 2014. Characterization of desmoglein expression in the normal prostatic gland. Desmoglein 2 is an independent prognostic factor for aggressive prostate cancer. *PLoS One* 9:e98786. 10.1371/journal.pone.0098786

Cai F, Zhu Q, Miao Y, Shen S, Su X, and Shi Y. 2017. Desmoglein-2 is overexpressed in non-small cell lung cancer tissues and its knockdown suppresses NSCLC growth by regulation of p27 and CDK2. *J Cancer Res Clin Oncol* 143:59-69. 10.1007/s00432-016-2250-0

Chen L, Liu X, Zhang J, Liu Y, Gao A, Xu Y, Lin Y, Du Q, Zhu Z, Hu Y, and Liu Y. 2018. Characterization of desmoglein 2 expression in ovarian serous tumors and its prognostic significance in high-grade serous carcinoma. *Int J Clin Exp Pathol* 11:4977-4986.

Fang WK, Gu W, Liao LD, Chen B, Wu ZY, Wu JY, Shen J, Xu LY, and Li EM. 2014. Prognostic significance of desmoglein 2 and desmoglein 3 in esophageal squamous cell carcinoma. *Asian Pac J Cancer Prev* 15:871-876. 10.7314/apjcp.2014.15.2.871

Jin R, Wang X, Zang R, Liu C, Zheng S, Li H, Sun N, and He J. 2020. Desmoglein-2 modulates tumor progression and osimertinib drug resistance through the EGFR/Src/PAK1 pathway in lung adenocarcinoma. *Cancer Lett* 483:46-58. 10.1016/j.canlet.2020.04.001

Ormanns S, Altendorf-Hofmann A, Jackstadt R, Horst D, Assmann G, Zhao Y, Bruns C, Kirchner T, and Knösel T. 2015. Desmogleins as prognostic biomarkers in resected pancreatic ductal adenocarcinoma. *Br J Cancer* 113:1460-1466. 10.1038/bjc.2015.362

Qin S, Liao Y, Du Q, Wang W, Huang J, Liu P, Shang C, Liu T, Xia M, and Yao S. 2020. DSG2 expression is correlated with poor prognosis and promotes early-stage cervical cancer. *Cancer Cell Int* 20:206. 10.1186/s12935-020-01292-x

Sun R, Ma C, Wang W, and Yang S. 2020. Upregulation of desmoglein 2 and its clinical value in lung adenocarcinoma: a comprehensive analysis by multiple bioinformatics methods. *PeerJ* 8:e8420. 10.7717/peerj.8420

Xu S, Huang S, Li D, Zou Q, Yuan Y, and Yang Z. 2020. Negative Expression of DSG1 and DSG2, as Prognostic Biomarkers, Impacts on the Overall Survival in Patients with Extrahepatic Cholangiocarcinoma. *Anal Cell Pathol* 2020:9831646. 10.1155/2020/9831646

Yashiro M, Nishioka N, and Hirakawa K. 2006. Decreased expression of the adhesion molecule desmoglein-2 is associated with diffuse-type gastric carcinoma. *Eur J Cancer* 42:2397-2403. 10.1016/j.ejca.2006.03.024
